# Poly (ADP-Ribose) Polymerase Inhibitor, ABT888, Improved Cisplatin Effect in Human Oral Cell Carcinoma

**DOI:** 10.3390/biomedicines9070771

**Published:** 2021-07-02

**Authors:** Irene Paterniti, Sarah Adriana Scuderi, Giovanna Casili, Marika Lanza, Marzia Mare, Raffella Giuffrida, Cristina Colarossi, Marco Portelli, Salvatore Cuzzocrea, Emanuela Esposito

**Affiliations:** 1 Department of Chemical, Biological, Pharmaceutical and Environmental Sciences, University of Messina, Viale Ferdinando Stagno D’Alcontres, 31-98166 Messina, ME, Italy; ipaterniti@unime.it (I.P.); sarahadriana.scuderi@unime.it (S.A.S.); gcasili@unime.it (G.C.); mlanza@unime.it (M.L.); salvator@unime.it (S.C.); 2 Istituto Oncologico del Mediterraneo, via Penninazzo 7, 95029 Viagrande, CT, Italy; marzia.mare@grupposamed.com (M.M.); cristina.colarossi@grupposamed.com (C.C.); 3 IOM Ricerca Srl, via Penninazzo 11, 95029 Viagrande, CT, Italy; raffaella.giuffrida@grupposamed.com; 4 Department of Biomedical and Dental Science, Morphological and Functional Images, University of Messina, via Consolare Valeria, 98125 Messina, ME, Italy; mportelli@unime.it

**Keywords:** oral cancer, human oral cell carcinoma, cisplatin, PARP inhibitors (PARPi), veliparib (ABT888), apoptosis

## Abstract

Cisplatin is one of the chemotherapeutic drugs used for the management of oral carcinoma, in which combined therapies are estimated to exert superior therapeutic efficacy compared with monotherapy. It is known that poly(ADP-ribosyl)ation is implicated in a multiplicity of cellular activities, such as DNA repair and cell death. Based on these, PARP inhibitors are used for the treatment of cancers; however, the capacity of PARP inhibitors associated to anti-cancer drugs have not been completely assessed in oral carcinoma. Here, we evaluated the effects of PARPi veliparib (ABT888) in combination with cisplatin on the survival of three human oral cancer cell lines HSC-2, Ca9-22 and CAL27 and we observed the effects of ABT888 alone or in combination with cisplatin on apoptosis and DNA damage repair mechanism. The results obtained showed that ABT888 induces a cytotoxicity effect on cell viability increasing the apoptotic pathway as well as DNA strand break; moreover, our results displayed the effects with cisplatin in a dose-dependent manner. Therefore, our results indicate PARP inhibitors as adjuvants for therapeutic strategy of oral cancer.

## 1. Introduction

Oral cancer represents one of the most widespread types of tumors that shows high morbidity, mortality [1] and metastasis [2] with an incidence of 450,000 new cases per year, affecting more men (15%) than women (6%) [3,4]. The incidence and mortality caused by this tumor show variability according to the geographic location in which it is diagnosed [4]. However, despite the progress in research and therapy, the survival rate has not improved significantly in the last few years, representing a continuing challenge for novel scientific studies [4]. Oral cancer is associated with genetic mutations, DNA damage and a marked infiltrating capacity [5]. Currently, oral cancer therapies include surgery as a treatment of choice, a chemotherapy approach with cisplatin and fluorouracil, or radiotherapy, or a combination of them as support therapy [6].

Although many drugs have been established as treatments for cancer, they commonly display cytotoxicity and side effects [7].

Specifically, in recent years, new treatments for oral cancer have been advanced; however, overall survival has not meaningfully diminished due to the alterations in tumor suppressor genes and variations in signaling pathways that lead to therapeutic resistance [8]. For this reason, it is warranted for continuing discovery for anti-oral cancer agents.

Poly (ADP-ribose) polymerases (PARPs) are a family of enzymes involved in several cellular processes such as DNA repair for cell survival, inflammatory responses and preservation of genome stability [9]. The PARP family consists of 17 members, which have different structures and functions within the cell [10]. The mechanism of action of PARP is related to its capacity to execute the post-translation modifications of proteins through the addition of PARP chains [11].

PARPs are able to catalyze reactions of Poly-ADP-ribosylation or mono-ADP-ribosylation [12]. Among PARPs, the most studied is PARP1, which plays a critical role in the DNA damage response and repair processes under stressful conditions, while PARP2 acts as a cofactor in gene transcription and promotes chromatin compaction [10].

Thus, given the key roles of PARPs, especially PARP1 and PARP2, in repairing cytotoxic chemotherapy-induced DNA damage, they have attracted attention as a new therapeutic strategy for cancer. The potential benefit of targeting the enzymatic activity of PARPs for therapeutic purposes is very high, supported also by its recent approval for clinical usage in the treatment of BRCA DNA repair-associated-mutated ovarian cancer [13,14]. Moreover, recent studies conducted in vivo and in vitro proposed that PARP inhibitors can enhance traditional chemotherapy in the treatment of cancer [15,16,17,18,19,20].

Among PARP inhibitors, more attention was given to Veliparib (ABT888), which, as well as other clinically relevant PARPi, targets DNA damage repair PARPs, mainly PARP1 and PARP2 [12].

An important study conducted by Yasukawa revealed the importance of the anti-tumor effects of PARP inhibitors used in combination with chemotherapeutic agents such as cisplatin thanks to their abilities to arrest cell cycle and decrease DNA repair activity through a G2 cell-cycle arrest-like effect in a dose and p53-dependent manner [21,22].

P53 is a tumor suppressor that responds to various stress signals by modulating specific cellular responses, including transient cell cycle arrest, cellular senescence and apoptosis, all of which are associated with tumor suppression [22]. However, recent studies have highlighted the role of p53 in modulating other cellular processes as well as metabolism, stem cell maintenance, invasion, metastasis and communication within the tumor microenvironment [21,22].

P53 deficiency as well as apoptosis alteration can enhance the initiation or progression of cancer [23], suggesting that studies on programmed cell death processes are needed to find new strategies for the treatment of oral tumors.

Thus, the combination of PARPi with anticancer treatments, including chemotherapy, molecular targeted agents, or immunotherapy, is certainly an encouraging method to maximize the effectiveness of tumor treatment, though they have been proven challenging due to overlapping toxicities with chemotherapy agents [24].

Therefore, on the basis of PARP functions and the key role of DNA damage and the apoptosis pathway on oral cancer [5,25,26], here we assessed the ability of Veliparib (ABT888), a selective PARPi, to enhance the anticancer effect of cisplatin in different cell cultures derived from oral cancers.

Such a combination is expected to exert a combinatorial effect, consequently resulting in superior therapeutic effects compared to drug administration alone, thus decreasing the side-effects and preventing drug resistance.

## 2. Material and Methods

### 2.1. Culture of Cell Lines Derived from Oral Carcinoma

Three cell lines, HSC-2 (human oral tongue squamous carcinoma cell line), Ca9-22 (human oral gingival squamous carcinoma cell line) and CAL27 (human tongue adenosquamous carcinoma cell line), were obtained from ATCC (Rockville, MD, USA) and were used in this study. The authors chose to use these three cell lines because they are the most studied cell lines in vitro for these tumors.

The growth medium for cells contained 10% fetal bovine serum (FBS) (Cultilab^®^, Campinas, Brazil), 100 U/mL penicillin and 100 μg/mL streptomycin (Sigma-Aldrich, St. Louis, MO, USA) in minimum essential Eagle’s medium (Sigma-Aldrich) for HSC-2 and Ca9-22 cells, and in Dulbecco’s Modified Eagle’s Medium (Sigma-Aldrich) for CAL27 cells. The cells were kept in an incubator at 37 °C with 5% CO_2_, and the change of growth medium was performed every three days.

### 2.2. MTT Assay

Cell viability was measured through MTT assay, as previously described by Esposito et al. [27]. HSC-2, Ca9-22 and CAL27 cells were pre-treated with increasing concentrations of ABT888 (15 μM, 30 μM and 60 μM) or cisplatin (5 μM, 10 μM, 15 μM and 20 μM) in order to assess high concentrations with high toxicity on cell viability. After 24 h, cells were incubated at 37 °C with MTT (0.2 mg/mL) for 1 h. The concentration of Veliparib 60 µM was chosen according to previous studies [28]. The medium was removed by aspiration and the cells lysed with DMSO (100 μL). The extent of reduction of MTT to formazan was quantified by measuring the optical density at 550 nm (OD550) with a microplate reader [29]. For another set of experiments, CAL27 cells were pre-treated with BIPV5 at a concentration of 50 μM 1h before treatments with 60 μM ABT888 and 15 μM cisplatin [30]. After 24 h, cells were incubated with MTT for 1h. The same procedure was conducted for CAL27 cells treated with PFT-α at a concentration of 30 μM [31].

### 2.3. RNA Isolation, cDNA Synthesis and Real-Time Quantitative PCR Amplification

Total RNA was isolated from the CAL27 cell line for RT-qPCR analysis using a Trizol Reagent Kit (Life Technologies, Monza, Italy), as previously described [32]. The first strand of cDNA was synthesized from 2.0 µg of total RNA using a high-capacity cDNA Archive kit (Applied Biosystems, Carlsbad, CA, USA). RT-qPCR was performed to evaluate the gene expression of Bcl-2, BAX, p53, caspase-3 and caspase-9. The amplified PCR products were quantified by measuring the calculated cycle thresholds (CT) of target genes and β-actin mRNA. β-actin mRNA was used as an endogenous control to allow for the relative quantification. After normalization, the mean value of the normal control target levels was chosen as the calibrator and the results were expressed as a fold change relative to normal controls. The oligonucleotide sequences of the used primers are reported in Table 1.

### 2.4. Western Blot Analysis

Western blot analysis was performed as previously described [33]. The cell monolayer was rapidly rinsed twice with ice-cold PBS and lysed in 1 mL of ice-cold lysis buffer. The lysis buffer contained 0.1 mM phenylmethyl sulfonyl fluoride, 2 mM EDTA, 25 mM-glycerophosphate, 0.1 mM sodium orthovanadate, 25 mM sodium fluoride, 5 µg of leupeptin, 0.2% Triton X-100 (Sigma-Aldrich Chemical Co, St Louis, MO, USA) and 0.3% Nonidet p-40 (Sigma-Aldrich Chemical Co) in 50 mM Tris-hydrochloride (Sigma-Aldrich Chemical Co)/150mM sodium chloride (pH 7.5). The lysates were centrifuged at 12,000× *g* at 4 °C for 15 min, and the supernatants were collected. Then, cell lysates were electrophoretically separated on an SDS-PAGE gel using a standard protocol. The proteins were then transferred to polyvinylidene fluoride (PVDF) membranes (IPVH00010; Millipore, Burlington, MA, USA). The membranes were blocked with 5% non-fat milk in Tris-buffered saline containing 0.1% Tween-20 (TBST) for 1 h at room temperature. The blots were then incubated with the following primary antibodies at 4 °C overnight (Table 2): anti-VEGF (1:500; Santa Cruz Biotechnology, Dallas, TX, USA, sc-7269), anti-PARP1 (1:500 Santa Cruz Biotechnology, Dallas, TX, USA, sc-8007), anti-PARG (1:500; Santa Cruz Biotechnology, Dallas, TX, USA), anti-Rad51 (1:500, Santa Cruz Biotechnology, Dallas, TX, USA, sc-398587), anti-γH2AX (1:500, Abcam), anti-Bax (1:500, Santa Cruz Biotechnology, Dallas, TX, USA, sc-7480), anti-Bcl2 (1:500, Santa Cruz Biotechnology, Dallas, TX, USA, sc-7382), anti-caspase-3 (1:500, Santa Cruz Biotechnology, Dallas, TX, USA, sc-56053), anti-p53 (1:500, Santa Cruz Biotechnology, Dallas, TX, USA, sc-126), anti-RIP1 (Cell Signaling Technology, Danvers, MA, USA Cat# 3493) and anti-MLKL (Abcam, Cambridge, UK, Cat# ab184718). Antibodies were diluted in PBS, 5% *w*/*v* nonfat dried milk and 0.1% Tween-20 (PMT) and incubated in the membranes at 4 °C, overnight. Subsequently, a secondary antibody was added to the membrane (1:2000, Jackson ImmunoResearch, West Grove, PA, USA) for 1 h at room temperature. Moreover, to ascertain that the blot was loaded with equal amounts of protein lysate, β-actin antibody (cytosolic fraction 1:500; Santa Cruz Biotechnology, Dallas, TX, USA) or lamin A/C (nuclear fraction 1:500, Santa Cruz Biotechnology, Dallas, TX, USA) were used as an endogenous control for protein expression of blots. Blots were analyzed as previously described [34].

### 2.5. Immunofluorescence Staining

AN immunofluorescence assay was performed on CAL27 cells, as previously described by Donaldson [23]. Cells were plated on glass cover slips and fixed in 4% paraformaldehyde in PBS (15 min at room temperature), permeabilized with 0.2% Triton X-100 and blocked with 0.1% BSA in PBS for 1 h at room temperature. Subsequently, cells were stained overnight (O/N) at 4 °C with primary antibody RAD51 (1:500, Santa Cruz Biotechnology, Dallas, TX, USA, sc-398587) and then incubated with the Alexa Fluor 488 goat anti-mouse antibody (green fluorescence) for 1 h at room temperature and shielded from light. DAPI (diamidino-2-phenylindole) was used to stain the nuclei (#940110 Vector Laboratories).

The images were taken with a confocal laser scanning microscope (CLSM) (Zeiss LSM700). The experiments were repeated at least three times to confirm results.

### 2.6. Xenograft Model

A Xenograft tumor model was established by subcutaneously inoculating 5 × 10^6^ CAL27 cells per tumor in 0.2 mL of Phosphate Buffered Saline (PBS) and 0.1 mL of Matrigel (BD Bioscience, Bedford, MA, USA) into BALB/c nude mice, as previously described by Yasukawa et al. [21]. Once the tumor diameter had reached 7 mm, the mice were randomly assigned to the following groups: (a) control (200 µL saline); (b) cisplatin (2 mg/kg per body weight, dissolved in 200 µL sterilized water) [35]; (c) ABT-888 (25 mg/kg, in a vehicle containing 0.85% NaCl adjusted to pH 4.0 using HCl); (d) combination (both cisplatin and ABT888). The chemicals were administered intraperitoneally every three days, five times [21].

The tumor size was monitored daily using a caliper and calculated as follows: V = W^2^ × L/2, where W and L represented the minor and major length. Three days after the last administration, all surviving mice were sacrificed, and tumors samples were processed for several analyses.

Animal experiments were in compliance with Italian regulations on the protection of animals used for experimental and other scientific purposes (DM 116192) as well as EU regulations (OJ of EC L 358/1 18 December 1986). The project identification code was 399/2019-PR released on May 24, 2019.

### 2.7. HE Staining

Haematoxylin and eosin (HE) staining was performed as previously described by Casili et al. [36]. Tumor sample sections of 7 μm thickness were processed and evaluated by a qualified histopathologist. All sections were studied using an Axiovision Zeiss microscope (Milan, Italy).

### 2.8. Materials

BAX Inhibiting Peptide V5 (Catalogue Number. B1436) and Pifitrin-α (Catalogue Number. 506132) were obtained from Sigma-Aldrich (St. Louis, MO, USA).

### 2.9. Statistical Analysis

All values are exposed as mean ± standard error of the mean (SEM) of “*n*” observations. Every analysis was executed three times, as an independent experiment. The results were examined using a one-way analysis of variance (ANOVA) followed by a Bonferroni post hoc test for multiple comparisons. A *p*-value of less than 0.05 was considered significant.

## 3. Results

### 3.1. Effects of ABT888 on Cell Viability

**Figure 1:**
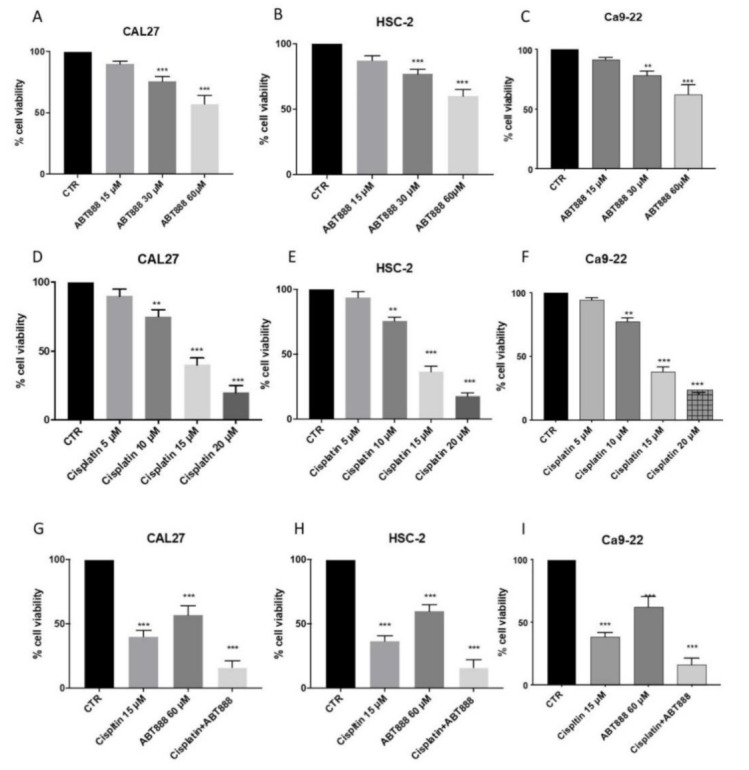
Effect of ABT888 on cell viability. Cell viability was assessed using MTT assay 24 h after treatment with ABT888 (15 μM, 30 μM, 60 μM) and cisplatin (5 μM, 10 μM, 15 μM and 20 μM). HSC-2, CAL27 and Ca9-22 cell lines showed a decrease in viability following ABT888 and cisplatin treatment, in particular at the lowest concentration (Figure A–F, respectively). Figures G, H and I showed that ABT888 at a concentration of 60 µM increased the cytotoxicity effect of cisplatin in all the cell lines. Data are representative of at least three independent experiments. (**A**) *** *p* < 0.001 vs. the control. (**B**) *** *p* < 0.001 vs. the control. (**C**) ** *p* < 0.01, *** *p* < 0.001 vs. the control. (**D**) ** *p* < 0.01, *** *p* < 0.001 vs. the control. (**E**) ** *p* < 0.01, *** *p* < 0.001 vs. the control. (**F**) ** *p* < 0.01, *** *p* < 0.001 vs. the control. (**G**) *** *p* < 0.001 vs. the control. (**H**) *** *p* < 0.001 vs. the control. (**I**) *** *p* < 0.001 vs. the control.

To evaluate the cytotoxic effects of ABT888 and cisplatin on the three cell lines derived from oral squamous cell carcinoma, we incubated HSC-2, CAL27 and Ca9-22 with different growing concentrations of ABT888 (15 μM, 30 μM, 60 μM) and cisplatin (5 μM, 10 μM, 15 μM and 20 μM) for 24 h. We performed MTT assays, and we observed weak toxicity effects at the lowest concentration of ABT888 (Figure 1A–C, respectively) and cisplatin in all three cell lines (Figure 1D–F, respectively); while the anti-proliferative effect increased in a concentration-dependent manner in all three cell lines (Figure 1A–F, respectively).

### 3.2. ABT888 Significantly Enhances the Cytotoxic Effect of Cisplatin in Oral Squamous Cell Carcinoma

Based on the results obtained on cell cytotoxicity, we saw that the best concentrations able to markedly reduce cell viability (± 50%) were 60 μM for ABT888 (Figure 1A–C) and 15 μM for cisplatin (Figure 1D–F) in all the cell lines used. Therefore, to evaluate if ABT888 enhanced the anti-proliferative effect of cisplatin, we decided to proceed with these two concentrations combined together. We performed an MTT assay, and we observed that when combined together ABT888 increased the cytotoxicity effect of cisplatin in all the cell lines (Figure 1G–I, respectively) compared with a single administration of ABT888 and cisplatin (Figure 1G–I, respectively).

### 3.3. Effects of ABT888 and Cisplatin on Apoptosis

**Figure 2:**
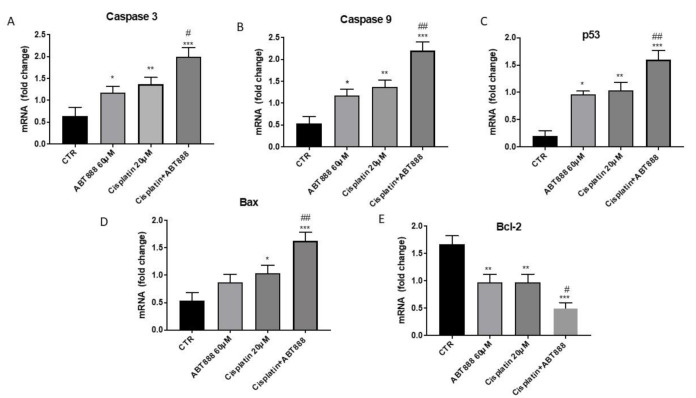
Effect of ABT888 and cisplatin on apoptosis pathway. Treatment with ABT888 and cisplatin increased Bax, caspase-3, caspase-9 and p53 mRNA expression in CAL27 cell line compared to single treatments and control groups, as shown in Figures A, B, C and D, respectively. Bcl2 mRNA expression was significantly reduced in the combinatory treatment as well as in the treatment of ABT888 and cisplatin alone compared to control group (**E**). Data are representative of at least three independent experiments. (**A**) * *p* < 0.05, ** *p* < 0.01, *** *p* < 0.001 vs. the control; # *p* < 0.05 vs. cisplatin group. (**B**) * *p* < 0.05, ** *p* < 0.01, *** *p* < 0.001 vs. the control; ## *p* < 0.01 vs. cisplatin group. (**C**) * *p* < 0.05, ** *p* < 0.01, *** *p* < 0.001 vs. the control ## *p* < 0.01 vs. cisplatin group. (**D**) * *p* < 0.05, *** *p* < 0.001 vs. the control; ## *p* < 0.01 vs. cisplatin group. (**E**) ** *p* < 0.01, *** *p* < 0.001 vs. the control; # *p* < 0.05 vs. cisplatin group.

Apoptosis is a crucial pathway involved in the development of tumors such as oral carcinoma. To verify if ABT888 improved the efficacy of chemotherapeutic agents to induce apoptosis in oral squamous carcinoma, we assessed RT-qPCR of pro- and anti-apoptotic proteins in the CAL27 cell line. We observed that a combinatory treatment of ABT888 and cisplatin considerably increased the expression of pro-apoptotic Bax, caspase-3, caspase-9 and p53 in the CAL27 cell line (Figure 2A–D), compared to single treatments (Figure 2A–D) and the control groups (Figure 2A–D). On the contrary, the expression of anti-apoptotic protein Bcl2 was significantly reduced in the combinatory treatment (Figure 2E) as well as in the treatment of ABT888 and cisplatin alone compared to the control group (Figure 2E).

### 3.4. Effect of ABT888 and Cisplatin on Apoptosis Pathway in CAL27, HSC-2 and Ca9-22 Cells

**Figure 3:**
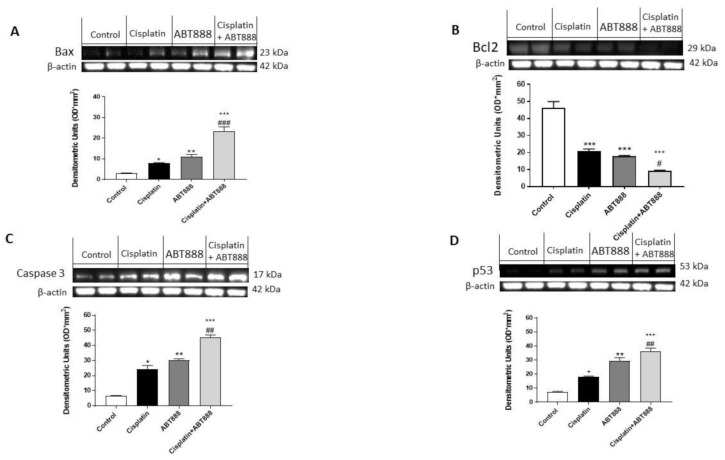
Effect of ABT888 and cisplatin on apoptosis pathway in CAL27 cell line. The blots revealed an increase in Bax, caspase-3 and p53 expression in the combination group of ABT888 and cisplatin, as shown in Figures A, C and D, respectively, compared to the single treatments and control group. On the contrary, the expression of Bcl2 was significantly reduced in the combinatory treatment of ABT888 and cis-platin, compared to the single treatments and control group, as shown in Figure B. (**A**) * *p* < 0.05, ** *p* < 0.01, *** *p* < 0.001 vs. the control; ### *p* < 0.001 vs. cisplatin group. (**B**) *** *p* < 0.001 vs. the control; # *p* < 0.05 vs. cisplatin group. (**C**) * *p* < 0.05, ** *p* < 0.01, *** *p* < 0.001 vs. the control; ## *p* < 0.01 vs. cisplatin group. (**D**) * *p* < 0.05, ** *p* < 0.01, *** *p* < 0.001 vs. the control; ## *p* < 0.01 vs. cisplatin group.

**Figure 4:**
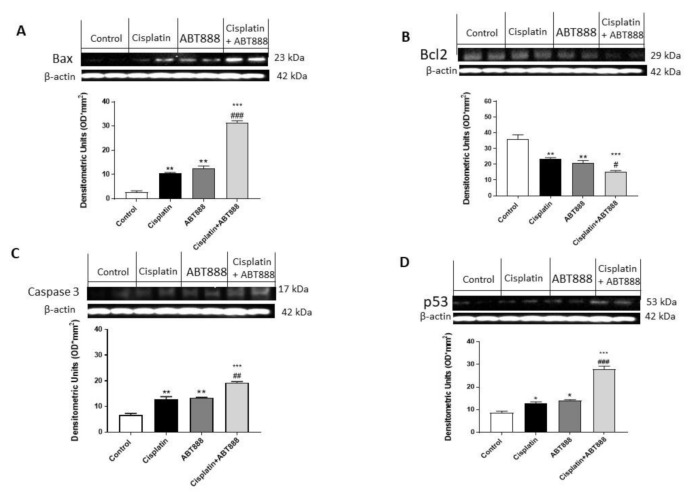
Effect of ABT888 and cisplatin on apoptosis pathway in HSC-2 cell line. The blots revealed an increase in Bax, caspase-3 and p53 expression in the group with the combinatory treatment of ABT888 and cisplatin (Figures A, C and D, respectively) compared to the single treatments and control group, whereas Bcl2 expression was significantly reduced in the combinatory treatment of ABT888 and cisplatin, compared to control group (Figure B). (**A**) ** *p* < 0.01, *** *p* < 0.001 vs. the control; ### *p* < 0.001 vs. cisplatin group. (**B**) ** *p* < 0.01, *** *p* < 0.001 vs. the control; # *p* < 0.05 vs. cisplatin group. (**C**) ** *p* < 0.01, *** *p* < 0.001 vs. the control; ## *p* < 0.01 vs. cisplatin group. (**D**) * *p* < 0.05, *** *p* < 0.001 vs. the control; ### *p* < 0.001 vs. cisplatin group.

**Figure 5:**
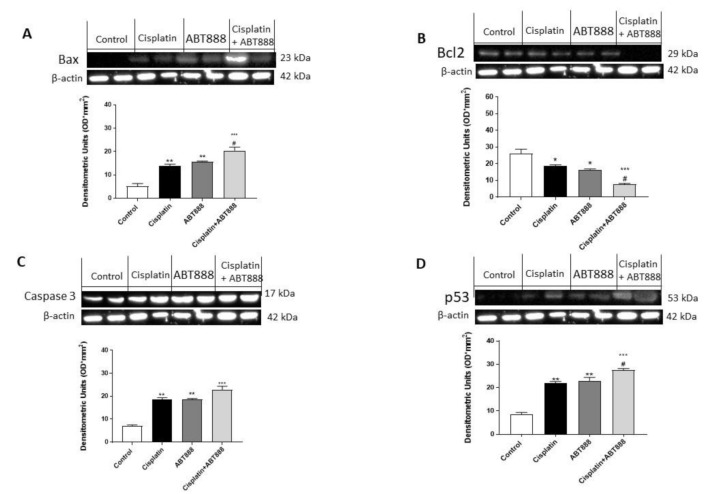
Effect of ABT888 and cisplatin on apoptosis pathway in Ca9-22 cell line. The blots revealed an increase in Bax, caspase-3 and p53 expression in the group with the combinatory treatment of ABT888 and cisplatin (Figures A, C and D, respectively) compared to the single treatments and control group, whereas Bcl2 expression was significantly reduced in the combinatory treatment of ABT888 and cisplatin, compared to control group (Figure B). (**A**) ** *p* < 0.01, *** *p* < 0.001 vs. the control; # *p* < 0.05 vs. cisplatin group. (**B**) * *p* < 0.05, *** *p* < 0.001 vs. the control; # *p* < 0.05 vs. cisplatin group. (**C**) ** *p* < 0.01, *** *p* < 0.001 vs. the control. (**D**) ** *p* < 0.01, *** *p* < 0.001 vs. the control; # *p* < 0.05 vs. cisplatin group.

To confirm the ability of ABT888 and cisplatin to modulate the apoptosis pathway, we decided to investigate Bax, Bcl2, p53 and caspase-3 expressions using Western blot analysis in the CAL27, HSC-2 and Ca9-22 cell lines. The results obtained in all three cell lines showed that the protein levels of BAX, p53 and caspase-3 were considerably increased in the combination group of ABT888 and cisplatin (Figure 3A,C,D, respectively; Figure 4A,C,D, respectively; Figure 5A,C,D, respectively) compared to the single treatments and control group. In contrast, Bcl2 expression was significantly reduced in the combinatory treatment ( Figure 3C, Figure 4C and Figure 5C, respectively) as well as in the treatment of ABT888 and cisplatin alone compared to the control group.

### 3.5. Evaluation of Mechanism of Action of ABT888

To assess the effects of cisplatin and ABT888 and its mechanism of action when associated together, we used Western blot analysis to analyze the protein levels of Poly(ADP-ribose) Glycohydrolase (PARG), which is involved in DNA repair [37], vascular endothelial growth factor (VEGF), γ-H2AX and RAD51 in the CAL27 cell line.

**Figure 6:**
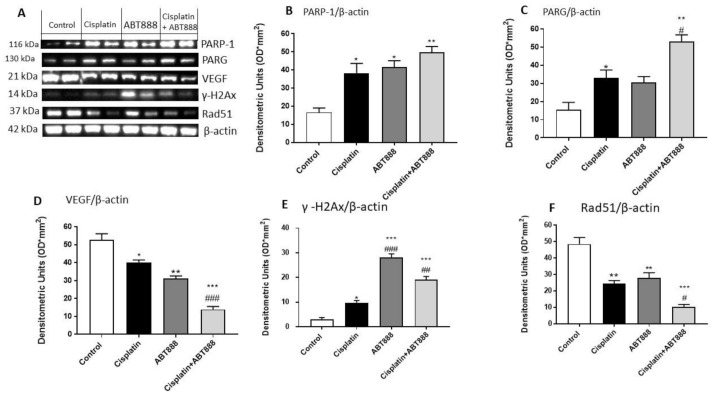
Effect of ABT888 and cisplatin on PARP-1, PARG, VEGF, γ-H2AX and RAD51 expression. The blots revealed an upregulation of PARP-1 and PARG expression in the combination group of ABT888 and cisplatin, as shown in Figures **A**, **B** and **C**, respectively, compared to the single treatments and control group. On the contrary, the expression of VEGF, γ-H2AX and RAD51 were significantly reduced in the combinatory treatment of ABT888 and cisplatin, compared to control group, as shown in Figures **D**, **E** and **F**, respectively. Data are representative of at least three independent experiments. (**B**) * *p* < 0.05, ** *p* < 0.01, vs. the control. (**C**) * *p* < 0.05, **p < 0.01, vs. the control; # *p* < 0.05 vs. cisplatin group. (**D**) * *p* < 0.05, ** *p* < 0.01, *** *p* < 0.001 vs. the control; ### *p* < 0.001 vs. cisplatin group. (**E**) * *p* < 0.05, *** *p* < 0.001 vs. the control; ## *p* < 0.01, ### *p* < 0.001 vs. cisplatin group. (**F**) ** *p* < 0.01, *** *p* < 0.001 vs. the control; # *p* < 0.05 vs. cisplatin group.

We observed that the protein levels of PARP-1 and PARG were considerably increased in the combination group of ABT888 and cisplatin (Figure 6A–C, respectively) compared to the single treatments and control group (Figure 6A–C, respectively). In contrast, the VEGF, γ-H2AX and RAD51 levels were significantly reduced in the combinatory treatment of ABT888 and cisplatin (Figure 6D–F, respectively) compared to the control group (Figure 6D–F, respectively).

### 3.6. Effect of ABT888 and Cisplatin on RAD51 Levels using an Immunofluorescence assay

**Figure 7:**
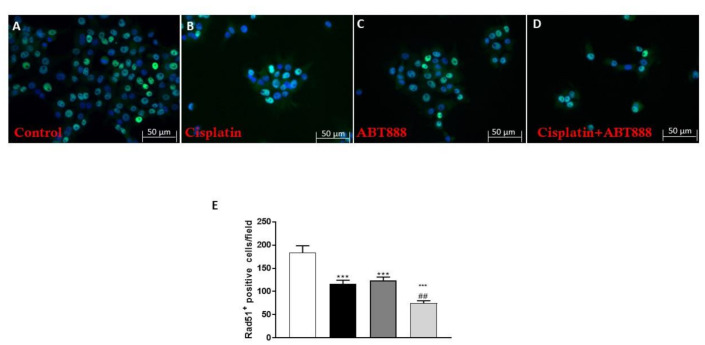
Effect of ABT888 and cisplatin on RAD51 levels on CAL27 cell line. Immunofluorescence assay revealed a marked expression of RAD51 expression in the control group (**A**), while the combinatory treatment with ABT888 and cisplatin (**D**) significantly reduced its expression compared to the single treatments (**B**,**C**). (**E**) *** *p* < 0.001 vs. the control; ## *p* < 0.01 vs. cisplatin group.

To confirm the results previously obtained on RAD51 expression using Western blot analysis, we decided to investigate the RAD51 levels using an immunofluorescence assay on the CAL27 cell culture. Our results showed an increase in RAD51 expression in the control group (Figure 7A), whereas the combinatory treatment of ABT888 and cisplatin (Figure 7D) was able to significantly reduce its expression compared to the single treatments (Figure 7B,C; see RAD51 positive cells score Figure 7E).

### 3.7. Evaluation of BIPV5 and PFT-α on CAL27 Cell Viability Alone or in Association with ABT888 and Cisplatin

**Figure 8:**
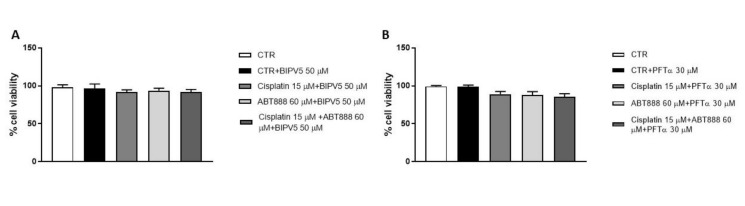
Effect of BIPV5 and PFT-α on CAL27 cell viability. MTT assay revealed that the apoptosis inhibitor BIPV5 and p53 inhibitor PFT-α did not reduce CAL27 cell viability in the control group as well as alone or in association with ABT888 and cisplatin, as shown in Figures **A** and **B**.

In this study, to confirm the involvement of the apoptosis process and p53 pathway in the mechanism of action of ABT888 in association with cisplatin, we conducted a cell viability assay on CAL27 cells using an inhibitor of apoptosis, BAX Inhibiting Peptide V5 (BIPV5), at a concentration of 50 µM [30] and an inhibitor of the p53 pathway, called Pifitrin-α (PFTα) at a concentration of 30 µM [31]. Our results showed that the pre-treatment with BAX Inhibiting Peptide V5 alone or in combination with ABT888 and cisplatin did not reduce CAL27 cell viability, as shown in Figure 8A. The same result appears for Pifitrin-α, as shown in Figure 8B, confirming that the apoptosis process and p53 pathway are involved in the mechanism of action of ABT888 and cisplatin.

### 3.8. Effect of ABT888 and Cisplatin on Necrosis Pathway

**Figure 9:**
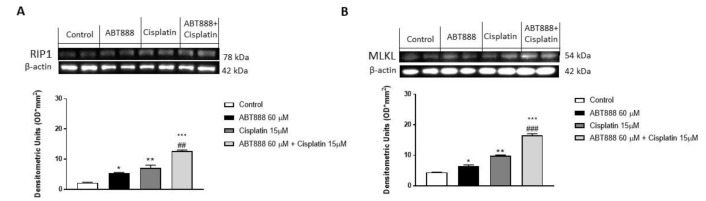
Effect of ABT888 and cisplatin on necrosis pathway in CAL27 cell culture. The association of ABT88 and cisplatin increased the expression of necrosis marker RIP1 and MLKL compared to the single treatments on CAL27 cells (**A**,**B**). Data are representative of at least three independent experiments. (**A**) * *p* < 0.05 vs. the control; ** *p* < 0.01 vs. the control; *** *p* < 0.001 vs. the control; ## *p* < 0.01 vs. cisplatin group. (**B**) * *p* < 0.05 vs. the control; ** *p* < 0.01 vs. the control; *** *p* < 0.001 vs. the control; ### *p* < 0.001 vs. cisplatin group.

On the basis of the obtained results, increasing PARP expression may indicate an increase in cell necrosis [38]. Therefore, in this study we decided to investigate the expression of necrosis markers as Receptor interacting protein-kinases-1 (RIP1) and Mixed lineage kinase domain-like protein (MLKL), involved in necrosis pathway [39,40] in the CAL27 cell culture. Our results showed an increase in RIP1 and MLKL expression in the combinatory group compared to the single treatments, suggesting an increase in the necrosis phenomenon thanks to their combinatory abilities (Figure 9A,B, respectively).

### 3.9. Effect of ABT888 and Cisplatin on Tumor Growth in CAL27-Xenograft Model

**Figure 10:**
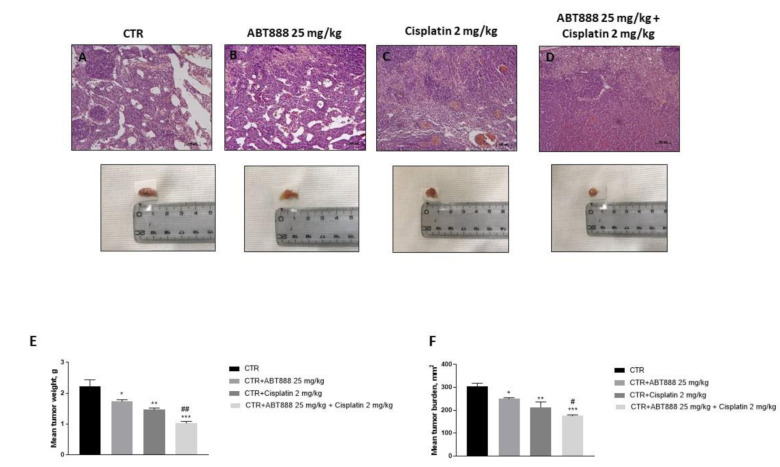
Effect of ABT888 and cisplatin on tumor growth. HE staining showed that the association between ABT888 and cisplatin significantly reduced tumor sections (**D**) much more than single components (**B**,**C**), compared to control group (**A**). Moreover, the association of ABT888 and cisplatin significantly reduced tumor weight as well as tumor burden (**E**,**F**). Data are representative of at least three independent experiments. (**E**) * *p* < 0.05 vs. the control; ** *p* < 0.01 vs. the control; *** *p* < 0.001 vs. the control; ## *p* < 0.01 vs. cisplatin group. (**F**) * *p* < 0.05 vs. the control; ** *p* < 0.01 vs. the control; *** *p* < 0.001 vs. the control; # *p* < 0.05 vs. cisplatin group.

The histological analysis revealed a marked subcutaneous mass in the control group, as well as an increase in the neutrophilic permeation, whereas the association between ABT888 and cisplatin showed a significant reduction in tumor sections much more than single components (Figure 10A–D). Moreover, in this study we observed that the association of ABT888 with cisplatin strongly inhibited tumor burden, as well as tumor weight (Figure 10E,F).

## 4. Discussion

Targeting DNA damage repair (DDR) signaling is a fast-expanding field for cancer therapy. DNA damage causes genomic instability in cells that require DDR for rescue. DDR signaling triggers the activation of repair protein transcription, hence, leading to overexpression of the related repair protein and activation of mechanisms for DNA repair [41]. PARP is an essential DNA repair protein, which has a significant function in the regulation of various DDR pathways, including Base Excision Repair (BER), Homologous Recombination (HR), classical and alternative Non-Homologous End Joining (NHEJ) and maintenance of replication fork stability [42]. In addition, PARP has been discovered to be overexpressed in several types of cancers, such as breast, ovarian and oral cancers, making inhibition of PARP activity a promising strategy for cancer therapy by disrupting PARP functions, thereby impairing the DDR pathways of cancer cells.

It has been recognized that PARP inhibition for the management of breast or ovarian cancers with BRAC1/2 mutations (deficiencies in the HR) is now a well-established approach, in fact over the past decade, four PARPi have been FDA-approved for clinical use as single agents.

In particular, previous studies reported that PARP inhibitors enhanced the anti-tumor effects of cisplatin, especially in breast cancer [43,44], as well as of temozolomide in Ewing’s sarcoma [45] or camptothecin in childhood neuroblastoma [46].

Thus, based on this evidence, in this study we evaluate if treatment with specific PARPi such as Veliparib (ABT888) could enhance the anti-tumor effect of cisplatin, when given in combination, for the treatment of oral carcinoma, representing a new therapeutic approach for this type of cancer.

The results obtained demonstrated that ABT888 revealed in vitro combinatorial effects with cisplatin in three cell lines derived from oral carcinoma, HSC-2, Ca9-22 and CAL27. In particular, we identified not only a selective anti-proliferative effect of ABT888 in a concentration dependent-manner, but we also observed that ABT888 potentiated the cytotoxic effect of cisplatin. Moreover, we observed that ABT888 exposure shows apoptosis-inducing effects on oral cancer cells, indicating its probable mechanism of action for its specific anti-proliferation effect.

Programmed cell death occurs thanks to two main mechanisms, specifically through two apoptotic signaling pathways, one defined as intrinsic and the other extrinsic [47]. The intrinsic pathway is also called the mitochondrial pathway and is controlled by the balance of two proteins, Bax and Bcl-2; apoptotic cell death is promoted by Bax, while Bcl-2 has the opposite role, it is in fact a protein with an antiapoptotic role [48]. On the other hand, the extrinsic pathway can lead to caspase activation and subsequent caspase-dependent apoptotic cell death. When activated, caspase-3 stimulates chromatin fragmentation that directs cell death [49].

Previous papers reported a synergetic effect of a PARPi (AZD2281) with cisplatin to cause arrest of the cellular cycle in G2/M and to decrease DNA repair activity in a dose and p53-dependent manner [22] in the HSC-2 cell line and S-phase arrest in the Ca9-22 cell line [50].

P53 is a tumor suppressor protein with a crucial anti-cancer role promoting cell cycle arrest, senescence and apoptosis in response to stress signals [51]. Currently, p53 is also considered a prognostic factor for esophageal cancer and oral carcinoma, suggesting the importance of new studies on programmed cell death processes to find new strategies for the treatment of oral tumors [51].

Therefore, in the present study, we proved that treatment with ABT888, and much more in combination with cisplatin, inhibited cell apoptosis in the CAL27-treated cell line, resulting in an increase in the mRNA expression of Bax and Bcl-2 downregulation via the p53 dependent pathway. Moreover, we observed that the levels of caspase-3 and 9 were significantly increased in ABT88 and the cisplatin-treated CAL27 cell line.

Moreover, to confirm the effect of ABT888 and cisplatin on the apoptosis pathway, we investigated Bax, Bcl2, caspase-3 and p53 protein expression in all three cell lines using Western blot analysis, showing that ABT888, and much more in combination with cisplatin, was able to increase the expression of pro-apoptotic Bax, p53 and caspase-3, while the expression of anti-apoptotic protein Bcl2 was significantly reduced.

Thus, in the present study we observed that ABT888, and much more in combination with cisplatin, was able to inhibit CAL27, HSC-2 and Ca9-22 tumor cell growth through the induction of apoptosis inducing p-53 and the caspase-dependent pathway, suggesting that ABT888 can activate the mitochondrial pathway of apoptotic cell death.

Moreover, since PARP is an SSB DNA repair enzyme, continuous exposure to PARP inhibitors increases the basal levels of SSBs, which may cause replication fork collapse and the formation of DNA double strand breaks (DSBs) [52,53]. DSBs are identified by phosphorylation of the core histone variant H2AX (forming γH2AX) marker of DNA damage.

Here, we observed that the level of γH2AX was increased in the combination group, although its expression was highest in the CAL-27 cell line treated with ABT888 alone, showing that the PARPi treatment could result in higher levels of DNA damages.

Moreover, here, we determined PARG, a glycohydrolase involved in DNA-damage repair [37], and PARP-1 expression to better understand tumor cells going toward apoptosis after drug treatments. The results obtained showed that either ATB888 treatment alone or in combination with cisplatin caused an important increase in DNA strand breaks according to PARG and PARP-1 increased expression that well correlated to γ-H2AX expression. These results indicate that, although the single treatments were capable of causing DNA damage, this mechanism is significantly increased in the combined treatment that could stimulate apoptosis in these cells to a greater extent.

Further analyses on the molecular basis of DNA breakdown and cytotoxicity were performed by examining the expression of the well-known DNA repair marker, DSB Rad51. Rad51 is a recombinase and an important molecule in the DNA repair pathway of homologous recombination (HR), which is recruited to the DNA break sites and forms separate nuclear foci. We found that the CAL-27 cell lines used in this study expressed high levels of Rad51, suggesting a recombinant functional repair capacity, which was also confirmed using an immunofluorescence assay. However, treatment with ABT888 alone, and much more with cisplatin, reduced the expression of Rad51, indicating a deficiency of human repair that could lead to more DSBs observed through the resulting increase in the expression levels of γ -H2AX. Moreover, to prove that ABT888 in association with cisplatin acts with an apoptosis-dependent and p53-dependent mechanism, we conducted a cell viability assay on a CAL27 cell treated with an inhibitor of apoptosis and an inhibitor of the p53 pathway. Our results showed that the apoptosis inhibitor BIPV5 and the p53 inhibitor PFT-α did not reduce CAL27 cell viability in the control group as well as alone or in association with ABT888 and cisplatin, confirming the hypotheses that ABT888 in association with cisplatin modulates apoptosis and the p53-dependent pathway. Furthermore, it cannot be excluded that in other cellular contexts, PARP inhibitors may act by involving other apoptotic mediators as well. Moreover, an increasing PARP expression may indicate cell necrosis; therefore, we demonstrated that the association of ABT888 and cisplatin significantly increased the expression of necrosis marker as RIP1 and MLKL on the CAL27 cell line, suggesting a promoting of the necrosis phenomenon thanks to the abilities of ABT888 and cisplatin.

Additionally, to confirm the data obtained in an in vitro model, we decided to conduct an in vivo xenograft model using CAL27 cells. The results obtained revealed that the association of ABT888 and cisplatin significantly reduced the tumor weight, tumor burden as well as the neutrophil infiltration much more the single treatments.

## 5. Conclusions

Thus, taken together, our results showed the therapeutic potential of PARPi in the treatment of oral cancer, suggesting that the combination of cisplatin and ABT888 could be a promising candidate for a treatment strategy. However, further investigations into the mechanism responsible for suppressing the DNA repair pathway of homologous recombination will be required. Meanwhile, this study would provide new insights into anticancer therapies, thus representing a new starting point.

## Figures and Tables

**Table 1:** Primer sequences used for TR-PCR.

Gene	Forward Primer 5’-3’	Reverse Primer 3’-5’
Bcl-2	GAGGATTGTGGCCTTCTTTGAG	AGCCTCCGTTATCCTGGATC
Bax	GGACGAACTGGACAGTAACATG	GCAAAGTAGAAAGGGCGGACA
p53	AGAGTCTATAGGCCCACCCC	GCTCGACGTAGGATCTGAC
Caspase-3	CTGAGGCATGGTGAAGAAGGA	GTCCAGTTCTGTACCACGGCA
Caspase-9	TGCGAACTAACAGGCAAGCA	GTCTGAACCTCTCTGGTTTGC
Β-actin	GACTTCGAGCAAGAGATGG	AGCACTGTGTGGCGTACAG

**Table 2:** Primary antibodies used for Western blot analysis.

Antibodies	Catalogue Number	Company	Source	Dilutions
**Bax**	sc-7480	Santa Cruz Biotechnology; Dallas, TX, USA.	Mouse	1:500
**Bcl2**	sc-7382	Santa Cruz Biotechnology; Dallas, TX, USA.	Mouse	1:500
**p53**	sc-126	Santa Cruz Biotechnology; Dallas, TX, USA.	Mouse	1:500
**Caspase-3**	sc-56053	Santa Cruz Biotechnology; Dallas, TX, USA.	Mouse	1:500
**PARP-1**	sc-8007	Santa Cruz Biotechnology; Dallas, TX, USA.	Mouse	1:500
**PARG**	sc-398563	Santa Cruz Biotechnology; Dallas, TX, USA.	Mouse	1:500
**Rad51**	sc-398587	Santa Cruz Biotechnology; Dallas, TX, USA.	Mouse	1:500
**VEGF**	sc-7269	Santa Cruz Biotechnology; Dallas, TX, USA.	Mouse	1:500
**γH2Ax**	sc-517336	Santa Cruz Biotechnology; Dallas, TX, USA.	Mouse	1:500
**β-actin**	sc-47778	Santa Cruz Biotechnology; Dallas, TX, USA.	Mouse	1:500
**MLKL**	ab184718	Abcam, Cambridge, UK.	Rabbit	1:500
**RIP1**	3493	Cell Signaling Technology, USA.	Rabbit	1:500

## Data Availability

The data presented in this study are available on request from the corresponding author.
